# Identifying the need for infection-related consultations in intensive care patients using machine learning models

**DOI:** 10.1038/s41598-024-52741-w

**Published:** 2024-01-28

**Authors:** Leslie R. Zwerwer, Christian F. Luz, Dimitrios Soudis, Nicoletta Giudice, Maarten W. N. Nijsten, Corinna Glasner, Maurits H. Renes, Bhanu Sinha

**Affiliations:** 1 Department of Health Sciences, University Medical Center Groningen, University of Groningen, Hanzeplein 1, 9713 GZ Groningen, The Netherlands; 2 Center for Information Technology, University of Groningen, Nettelbosje 1, 9747 AJ Groningen, The Netherlands; 3 Department of Medical Microbiology and Infection Prevention, University Medical Center Groningen, University of Groningen, Hanzeplein 1, 9713 GZ Groningen, The Netherlands; 4 Department of Critical Care, University Medical Center Groningen, University of Groningen, Hanzeplein 1, 9713 GZ Groningen, The Netherlands

**Keywords:** Infectious diseases, Microbiology, Antimicrobials, Clinical microbiology, Infectious-disease diagnostics, Computer science, Information technology, Scientific data, Statistics

## Abstract

Infection-related consultations on intensive care units (ICU) have a positive impact on quality of care and clinical outcome. However, timing of these consultations is essential and to date they are typically event-triggered and reactive. Here, we investigate a proactive approach to identify patients in need for infection-related consultations by machine learning models using routine electronic health records. Data was retrieved from a mixed ICU at a large academic tertiary care hospital including 9684 admissions. Infection-related consultations were predicted using logistic regression, random forest, gradient boosting machines, and long short-term memory neural networks (LSTM). Overall, 7.8% of admitted patients received an infection-related consultation. Time-sensitive modelling approaches performed better than static approaches. Using LSTM resulted in the prediction of infection-related consultations in the next clinical shift (up to eight hours in advance) with an area under the receiver operating curve (AUROC) of 0.921 and an area under the precision recall curve (AUPRC) of 0.541. The successful prediction of infection-related consultations for ICU patients was done without the use of classical triggers, such as (interim) microbiology reports. Predicting this key event can potentially streamline ICU and consultant workflows and improve care as well as outcome for critically ill patients with (suspected) infections.

## Introduction

Critically ill patients frequently require clinical care on intensive care units (ICU). These patients often have complex medical conditions and often display a dynamic clinical course. Providing appropriate ICU care necessitates highly skilled ICU personnel, operating in shifts 24/7, as well as technical infrastructure, e.g. for continuous patient monitoring and mechanical ventilation. Complexity is further increased since many interventions are time-critical, requiring prompt action in response to alteration of a patient’s clinical situation. An example is rapid initiation of supportive care and appropriate antimicrobials for patients with (suspected) sepsis^1^. In parallel, often other medical specialties are required in a consulting role. For patients with a (suspected) infection, additional input on clinical microbiology (CM), infectious diseases (ID), or antimicrobial stewardship (AMS) is usually consultation-based. A positive impact of infection-related consultations on patient care and outcome has been shown in several scenarios. A systematic review and meta-analysis of observational studies demonstrated an improvement of quality of care and reduction of mortality in patients with *Staphylococcus aureus* bacteraemia through infection-related consultations^2^. Furthermore, in a cohort study including 128 patients with *Pseudomonas aeruginosa* bacteraemia, patients receiving infection-related consultations had reduced 30-day mortality, improved source control and a treatment that was better in accordance with the guidelines compared to patients who did not receive these consultations^3^. Face-to-face/bedside visits showed an improved quality of care for bacteraemic patients compared to phone-based consultations alone^4,5^. Consultations in a face-to-face or handshake approach were also found to be positively associated with quality of care in antimicrobial stewardship studies^6,7^. However, the optimal timing and trigger for infection-related consultations are difficult to set. Most often, triggers for consultation are event-based (e.g., positive culture or clinical events), thus reactive rather than proactive. Another approach is planning at fixed time points, as in the examples above, thus not ideally suited for highly dynamic clinical situations.

As a consequence of the complex and dynamic clinical situation, rapid and flexible interventions are required which also includes infection-related consultations. In non-ICU patients, these requirements are much less applicable and consequently routine infection-related consultations at predefined time points are easier to implement. The timeliness of a consultation is a key element of high-quality consultations^8^. Timeliness can be facilitated and improved through automated process support. Automatic notification from the microbiology department to trigger CM/ID consultations can result in a significantly decreased delay to consultation and improved quality of care^9,10^. However, while a laboratory-triggered or pharmacy-triggered approach is usually easy to implement and already part of the routine in many settings^6^, automating the identification of patients in need of a consultation on the ICU side is more challenging.

The identification of the need for a consultation triggered by the ICU team is based on a plethora of available information. A patient’s history, diagnostic results, vital signs, clinical examination, clinical risk scores, and the patient’s clinical course over time produce large numbers of data points. All this information is taken into account by the ICU team with their expertise and experience for any clinical decision-making process. The framework of this process and its complexity is under research, and the list above is far from complete^11–14^. On average intensivists make 8.9 decisions per patient per day^13^. The decision to initiate an external, infection-related consultation could, for a simplified example, be driven by a combination of several factors: changes in infection-related laboratory results such as an increase in C-reactive protein (CRP), deteriorating vital signs (e.g. increase in heart rate and decrease in blood pressure), results in imaging suggesting the presence of an infection (e.g. pulmonary infiltrates on chest x-ray images), and the lack of a (timely) response to administered antimicrobial therapy. While individual clinical reasoning of ICU team members is more difficult to reflect and store in electronic health records (EHR), large numbers of data points, such as the examples above, are generated for ICU patients every second. This data could be used to automate, inform, and support the process of notification and triggering of infection-related consultations.

Machine learning, using statistical tools to identify patterns in large amounts of data, has the potential to support early identification and triggering of infection-related consultations. The use of machine learning in infectious diseases and microbiology is increasing. It covers a wide range of infection-related aspects and is often based on ICU data^15–18^. A potential application of machine learning was established for detecting bloodstream infections, bacteremia and sepsis or post-surgery complications^15–18^. Recently, successful prediction of survival of ICU patients using imaging and non-imaging routine clinical data by ML models has been reported^19^. However, the notification, initiation, or triggering of infection-related consultations has not yet been the subject of machine learning research. Therefore, this study aimed at identifying the need for an infection-related consultation in ICU patients several hours in advance by developing a machine learning model using data routinely collected in the EHR.

## Methods

### Study setting

This study was performed at the University Medical Center Groningen (UMCG), a 1339-bed academic tertiary care hospital in the North of the Netherlands. Ethical approval was obtained from the institutional review board (Medical Ethical Committee) of the UMCG, and informed consent was waived by this committee due to the retrospective observational nature of the study (METc 2018/081). The study was performed in accordance with relevant local, national and international guidelines/regulations. It included patients admitted to the 42-bed multidisciplinary adult critical care department with several ICUs at the UMCG. All patients were admitted between March 3, 2014, and December 2, 2017, based on the use and database availability of the local EHR system during this time. Patients were included in this study if they were registered in this EHR system, did not object to their use of data in the UMCG objection registry, and were at least 18 years old at the time of ICU admission. All patient data was anonymised prior to analysis.

### Infection-related consultations

Consultations analysed in this study were performed by clinical microbiologists and ID specialists of the hospital’s Department of Medical Microbiology and Infection Prevention. This department is responsible for all microbiological and infection control/prevention services at the UMCG. It offers a full spectrum of state-of-the-art diagnostic procedures for rapid, precise, and patient-specific diagnosis of infections. The department provides consulting assistance to the ICU in the form of a dedicated clinical microbiologist/ID specialist with 24/7 availability. Consultations at the ICU were triggered through various ways: (1) clinically-triggered by request from intensivists, (2) laboratory-triggered by pertinent diagnostic findings, (3) routine monitoring of newly admitted patients by a clinical microbiologist/ID specialist, or (4) by regular in-person participation in clinical rounds at the ICU which can also result in proactive “by-catch” consultations. In addition, the clinical microbiologist/ID specialist in charge attends the daily ICU multidisciplinary patient board. All consultations were recorded in the local database at the Department of Medical Microbiology and Infection Prevention.

### Data extraction and processing

Data was extracted from local EHR systems. The ICU EHR system comprised 1909 raw features (variables) covering all laboratory results, point-of-care test results, vital signs, line placements, and prescriptions. Demographic and administrative patient information was extracted from an administrative hospital database. Infection-related consultation timestamps per patient were obtained from the laboratory information system (LIS) at the Department of Medical Microbiology and Infection Control. All data were cleaned to remove duplicates, extract numeric values coded in text, and standardize time stamps where appropriate.

Laboratory and point-of-care test results were included if at least one data point per feature was available in at least 30% of all patient admissions. This resulted in 121 features. Numeric features were cleaned to exclude outliers (physiologically impossible values), which typically indicated faulty tests or missing test specimens. All feature timestamps were available at the minute level. For numeric features the mean per minute was taken if more than one data point per minute was available. No double entries per minute were observed for categorical values. Categorical values indicating missing specimens or other error messages (e.g., “wrong material sent”) were transformed to missing values. Laboratory features were re-coded to indicate if values fell in between feature-specific reference ranges based on available, local reference values.

Raw vital signs were cleaned for outliers (e.g., systolic arterial blood pressure smaller than diastolic arterial blood pressure), which usually indicated faulty measurements (e.g., through kinked lines). Line placement data was transformed to a binary feature indicating the presence or absence of an intravenous or arterial line per minute and line type. Prescription data were filtered to include prescriptions of the categories: antimicrobials (identified through agent specific codes in the EHR), blood products, circulatory/diuresis, colloids, crystalloids, haemostasis, inhalation, cardiopulmonary resuscitation, and sedatives. All prescriptions were transformed to binary features indicating the presence of a prescription per minute, agent, and type of administration. Additional binary features were introduced indicating the presence of a prescription per prescription category and type of administration. Dosing was available but not in standardized format and therefore omitted. Selective digestive decontamination (SDD) is a standard procedure in our hospital and was thus indicated by a distinct variable to avoid confusion with antimicrobial agents for other purposes^20^.

To prevent sparseness, all time dependent data were aggregated per 8-h intervals (arbitrary virtual shifts) of the respective ICU stay. We chose to aggregate the data to arbitrary virtual 8-h shifts to mimic clinicians’ behaviour in reviewing what took place the previous shift before making medical decisions in the current shift. The aggregation was done by taking the mean value over the 8-h interval or taking the occurrence of an event in the case of dichotomized features. For laboratory values, the last observation was used, which we have operationally defined as clinically the most relevant observation. Supplementary Table 1 shows an overview of the missing data after 8-h aggregation. Of note, missing data for laboratory and point of care tests, prescriptions and line placements are either truly missing, or missing because the action was not performed. However, due to the importance of having correct data for these features for clinical reasoning, we assumed that these features were missing for the latter reason. Missing values were filled with the last available data point before the missing value^21^. This carry-forward imputation process was used to mimic common physician’s behaviour. Remaining missing numeric values were imputed using the median of the feature’s overall distribution. Near zero variance predictors (features not showing a significant variance) were dropped from the dataset at a ratio of 95/5 for the most common to the second most common value. Each patient’s stay was treated as an independent stay, but a readmission feature was introduced, indicating how often the patient was previously admitted during the study period.

**Figure 1:**
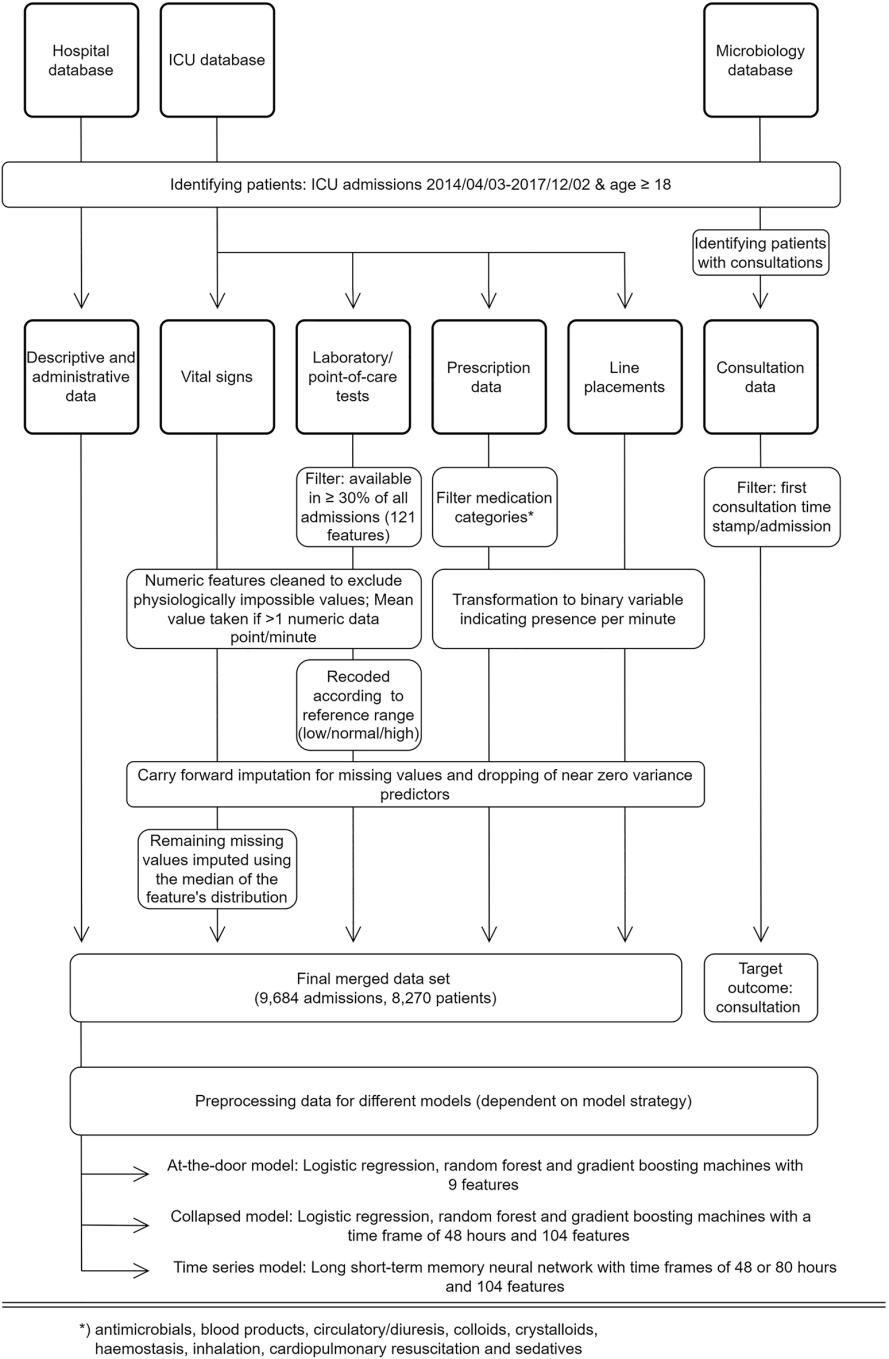
Study design and data processing for three different data sources (hospital database, ICU database, and medical microbiology database). Note: (interim) microbiology reports were not included in the modelling process. Standard data cleaning processes are not shown (cf. main text in “Methods”).

Consultation data included the time of documentation of infection-related consultations for patients admitted to the ICU. These include a variable delay between action and documentation, and a non-standardized free text documentation. The type of consultation (i.e., via phone or in person) was not available. Data were filtered to identify the first consultation time stamp per patient and admission. This point in time formed the targeted outcome of this study. Subsequent consultations were disregarded as they involve follow up on the clinical course of the initially presented problem and are therefore largely clinically predictable. Moreover, predictions of later consultations will consequently lead to only nominal time gain. The final dataset used in the modelling process comprised 104 features (see Supplementary Table 2). An overview of the study design including the data processing is shown in Fig. 1.

### Cohort investigation

Descriptive analyses were stratified by consultation status (consultation vs. no consultation). Baseline patient characteristics were assessed and compared with Fisher’s exact test for categorical features and Student’s t-test for continuous features. Logistic regression was used to create an explanatory model for infection-related consultations using the baseline features. Odds ratios with 95% confidence interval were used in the result presentation.

### Modelling process

Three different modelling approaches were used and evaluated in this study to predict an infection-related consultation. Each model made one prediction per patient and admission (i.e., sequence-to-one classification). The first approach (*at-the-door* model) used static patient features available at the time of admission: gender, age, body mass index, weekend admission, mechanical ventilation at admission, referring specialty, planned admission, readmission, and admission via the operation room. We used two outstanding powerful ensemble models, random forest (RF)^22^, and gradient boosting machines (GBM)^23^, and a commonly used generalised linear model (i.e. logistic regression)^24^ to predict the need for an infection-related consultation at any time during a patient’s stay in the ICU. Predictions were made at the time of the admission to the ICU.

The second modelling approach (*collapsed* model) also used LR, RF, and GBM and additional time-dependent procedural features measured during the ICU stay, such as the presence of medication, lines, performed diagnostics, and vital signs (see Supplementary Table 2) to predict a consultation up to eight hours (one arbitrary virtual shift) in advance. Since LR, RF, and GBM are not suited to handle raw longitudinal data, an additional pre-processing step was required. To enable the inclusion of time dependent features in the model, longitudinal data were aggregated to predict the target event (infection-related consultation) by calculating the mean, the standard deviation, minimum, maximum and trend over the available data. In the case of dichotomised variables, time series were aggregated by taking the proportions over time. Aggregation was done over a time span of 48 h. In case that patients stayed less than 48 h on the ICU all available data were aggregated. The arbitrary virtual shift in which the consultation took place was not included in the aggregation. The aggregation process led to missing values for the standard deviations and trends in the case that patients had less than two observations prior to a consultation. The RF and GBM models, as a standard feature, are able to handle these missing values. For the LR all observations with missing values were removed prior to the analysis (3405 admissions). This led to a dataset with 6279 admissions for the LR. For completeness the RF and GBM models were trained and evaluated on a dataset with and without missing values.

The aggregated data were used to predict the need for a consultation in the next arbitrary virtual shift (i.e., between zero and eight hours in advance). For patients receiving a consultation the 48 h of aggregated data before the shift in which the consultation took place were used to predict a consultation in the next arbitrary virtual shift. To create an unbiased ending point for patients who did not receive a consultation we used 48 h of aggregated data up to a random point during their ICU stay to predict the event (i.e., infection-related consultation or not) in the next arbitrary virtual shift. This ending point was randomly taken between one arbitrary virtual shift after admission up to one arbitrary virtual shift before discharge.

The third approach used a long short-term memory neural network (LSTM) to model the target outcome (*time-series* model). LSTM is an artificial recurrent neural network with the advantage to include memory and feedback into the model architecture. This *time-aware* nature (and its similarity to clinical reasoning) in addition to previous reports on the beneficial use of LSTM in the field of infection management with EHR built the background for the choice of this methodology^25–27^. An LSTM has the advantage that the data did not need to be aggregated to be used in the model, but all available information could be used without the need for additional feature pre-processing. Following the same approach as for the *collapsed* model, the LSTM model included all available features (see Supplementary Table 2). To prepare the model for the LSTM, data were split into arbitrary virtual 8-h shifts of a given ICU stay. Each of these arbitrary virtual shifts represented one time step in the model. Two different LSTM models were trained. First and similar to the *collapsed* approach, a time frame of 48 h (i.e., six virtual arbitrary shifts) was used to predict the occurrence of an infection-related consultation in the next virtual arbitrary shift. Finally, a second LSTM model used a time frame of 80 h (i.e., ten virtual arbitrary shifts) to predict infection-related consultations in the next virtual arbitrary shift. The virtual arbitrary shift in which the consultation took place was not included in the data used to make the prediction. The different shifts (i.e., six or ten virtual arbitrary shifts) of the data were used to predict an infection-related consultation in the seventh or eleventh virtual arbitrary shift, respectively. To create an unbiased endpoint for patients who did not receive a consultation we used the different shifts (i.e., six or ten virtual arbitrary shifts) of the data up to a random point during their ICU stay to predict the event (i.e., infection-related consultation or not) in the next virtual arbitrary shift. This random point was taken between one virtual arbitrary shift after admission up to one arbitrary virtual shift before discharge. Data for patients who had length of stay shorter than six or ten arbitrary virtual shifts were padded to ensure inputs of the same size.

The *time-series* models were trained with and without the use of standard deviations of each time dependent feature within each arbitrary virtual shift. Laboratory values were an exception to this as most laboratory results were less frequent than once per arbitrary virtual shifts. In that case, the standard deviation is undefined leading to many missing values. Therefore, no standard deviations were taken for laboratory values.

### Training, optimization, and evaluation of the models

The data was randomly split into a train and a test set using an 80-20 split. All models were trained and evaluated on the same train and held-out test set. Fisher’s exact test for categorical features and Student’s t-test for continuous features were performed for the target and all baseline characteristics on the train and held-out test set to ensure similar baseline characteristics. Model performances were evaluated and compared using the area under the receiver operating curve (AUROC) and the area under the precision recall curve (AUPRC).

All machine learning models were trained with and without addressing class imbalance. For the *at-the-door* and *collapsed* models class imbalance was addressed using oversampling. For the *time-series* models class imbalance was addressed using higher class weights for the minority class. All models were evaluated using tenfold cross validation. Confidence intervals of the performance on the validation set were calculated using the results on the different validation folds. A random grid search was used to select the optimal hyperparameters for the *at-the-door* models as well as the *collapsed* models. For the RF we used a thousand trees and for the GBM we used 5000 trees. However, we tested if 2000 or 6000 trees increased performance. An early stopping was applied to both models, where the moving average of the ten best stopping rounds was compared to the most recent stopping round and if performance did not increase substantially no trees were added (i.e., stopping_rounds = 10). For both the RF as well as the GBM a grid search was performed on the minimum number of observations in a leaf to split (min_rows), the maximum tree depth (max_depth) and the number of observations per tree (sample_rate). For RF we additionally searched for the optimal value for the number of features to sample at each split (mtry). Moreover, for the GBM a grid search was performed on the number of features per tree (col_sample_rate), the learning rate (learn_rate) and the reduction in the learning rate after every tree (learn_rate_annealing). The LSTM model was manually tuned on the validation folds by adding layers, adjusting the number of units, the drop out and batch size. The effects of these model adjustments were monitored using learning curves per epoch displaying the train and validation performance. Calibration plots, calibration intercepts and slopes, scaled Brier score, decision curve analysis, and feature importance plots were analysed for all best performing models per modelling approach. Finally, we analysed the model performance of all best performing models per modelling approach on sub-populations with different characteristics (e.g. planned admissions, post-operative patients).

All data processing and analyses were performed using tidyverse and H2O in R and numpy, pandas, caret, scikit-learn, keras, and tensorflow in Python^28,29^. The data is registered in the Groningen Data Catalogue (https://groningendatacatalogus.nl). The study followed the Transparent Reporting of a Multivariable Prediction Model for Individual Prognosis or Diagnosis (TRIPOD) statement (template available in Supplementary Table 3)^30^.

## Results

### Patient population

In total, 8270 unique patients and 9684 admissions were included in the study (Table 1).

**Table 1:** Patient characteristics.

	Consultation (N = 759)	No consultation (N = 8925)	Total (N = 9684)	p-value*
Gender				0.786
Female	290 (38.2%)	3461 (38.8%)	3751 (38.7%)	
Male	496 (61.8%)	5464 (61.2%)	5933 (61.3%)	
Age at admission (years)				0.017
Mean (± SD)	61.6 (14.6)	60.3 (15.3)	60.4 (15.3)	
Range	19–92	18–101	18–101	
Number of readmissions				
Mean (± SD)	0.2 (0.5)	0.1 (0.4)	0.1 (0.4)	< 0.001
Range	0–4	0–6	0–6	
Admission via operation room				< 0.001
Not via OR	508 (66.9%)	3183 (35.7%)	3691 (38.1%)	
Via OR	251 (33.1%)	5742 (64.3%)	5993 (61.9%)	
Planned admission				< 0.001
No	635 (83.7%)	4345 (48.7%)	4980 (51.4%)	
Yes	124 (16.3%)	4580 (51.3%)	4704 (48.6%)	
Length of ICU stay (days)				< 0.001
Mean (± SD)	9.0 (10.7)	3.6 (5.4)	4.0 (6.2)	
Median	5	2	2	
Range	1–119	1–96	1–119	
Hospital mortality (ICU)				< 0.001
Died	348 (45.8%)	365 (4.1%)	713 (7.4%)	
Survived	411 (54.2%)	8560 (95.9%)	8971 (92.6%)	

*Comparing consultation vs. no consultation; Fisher’s exact test for categorical features; Student’s t-test for continuous features.

### Consultations

**Figure 2:**
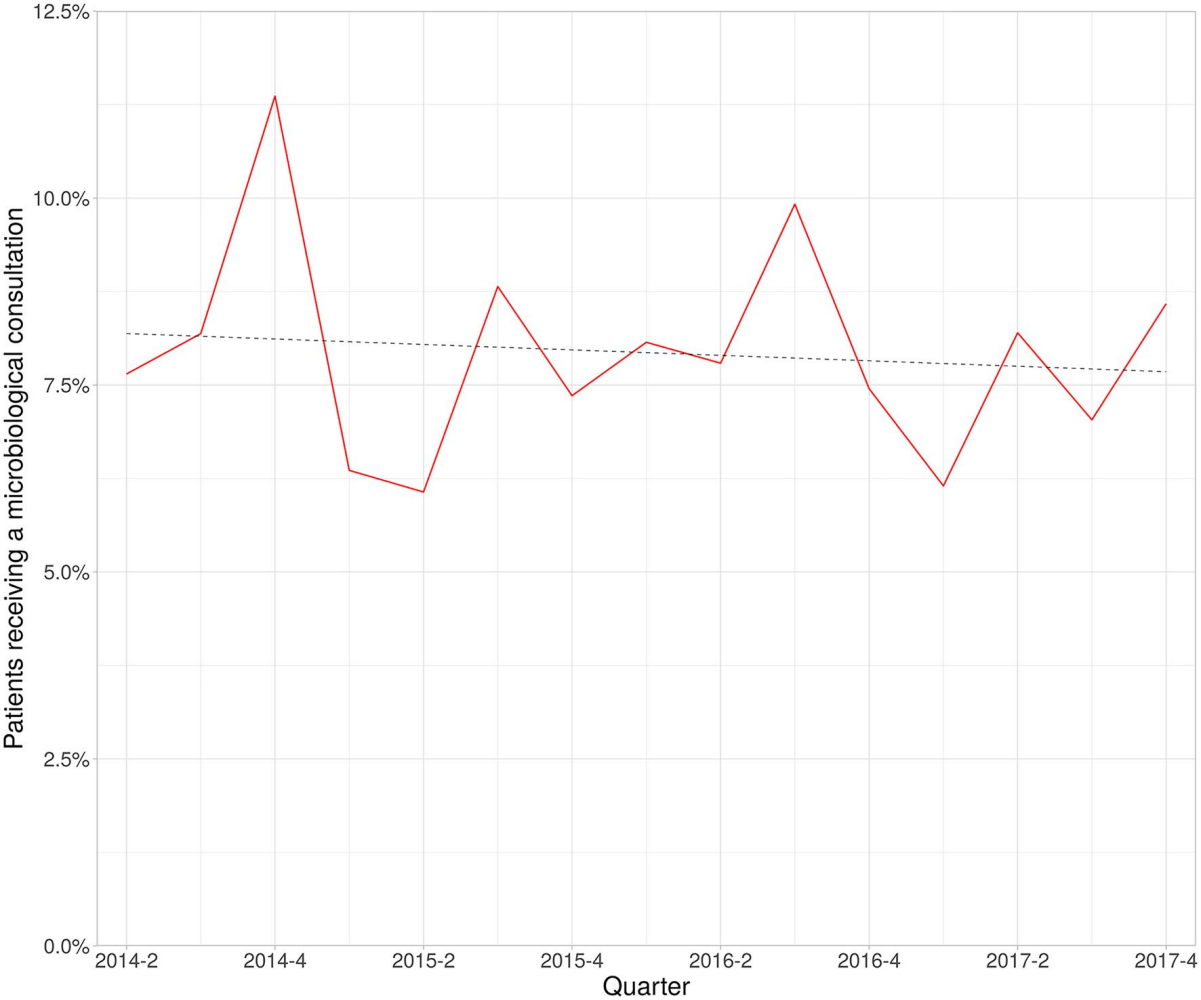
Proportion of admissions with an infection-related consultation per quarter. No significant change in trend line (dashed) using a linear regression model.

The proportion of patients with a consultation did not show a significant trend over time and centred around the mean of 7.9% (SD: 1.4%) of all patients over all quarters of the study period (Fig. 2). An explanatory multiple logistic regression analysis using available basic patient and administrative features was performed to identify characteristics of the consultation cohort. Several features showed a significant positive association with consultations: age, mechanical ventilation at ICU admission, and number of readmissions (Table 2). A significant negative association with consultations was found for admission via the operation room and planned admissions, reflecting elective, post-operative care for this patient cohort.

**Table 2:** Multiple logistic regression model for infection-related consultations at the ICU.

Variable	Univariate analysis		Multivariate analysis*	
	OR (95% CI)	p-value	OR (95% CI)	p-value
Weekend admission	1.66 (1.38–2.00)	< 0.001	0.89 (0.73–1.07)	0.220
Gender				
Female	Reference	N/A	Reference	N/A
Male	1.02 (0.88–1.19)	0.757	1.03 (0.88–1.21)	0.691
Age (years)	1.01 (1.00–1.01)	0.022	1.01 (1.00–1.01)	< 0.001
Body Mass Index	1.01 (1.00–1.02)	0.204	1.01 (1.00–1.02)	0.102
Mechanical ventilation at ICU admission	1.24 (1.07–1.45)	0.004	1.66 (1.41–1.95)	< 0.001
Number of readmissions	1.43 (1.24–1.64)	< 0.001	1.21 (1.04–1.40)	0.014
Admission via operation room	0.27 (0.23–0.32)	< 0.001	0.47 (0.39–0.56)	< 0.001
Planned admission	0.19 (0.15–0.23)	< 0.001	0.27 (0.22–0.34)	< 0.001

*OR* odds ratio, *CI* confidence interval.

**Figure 3:**
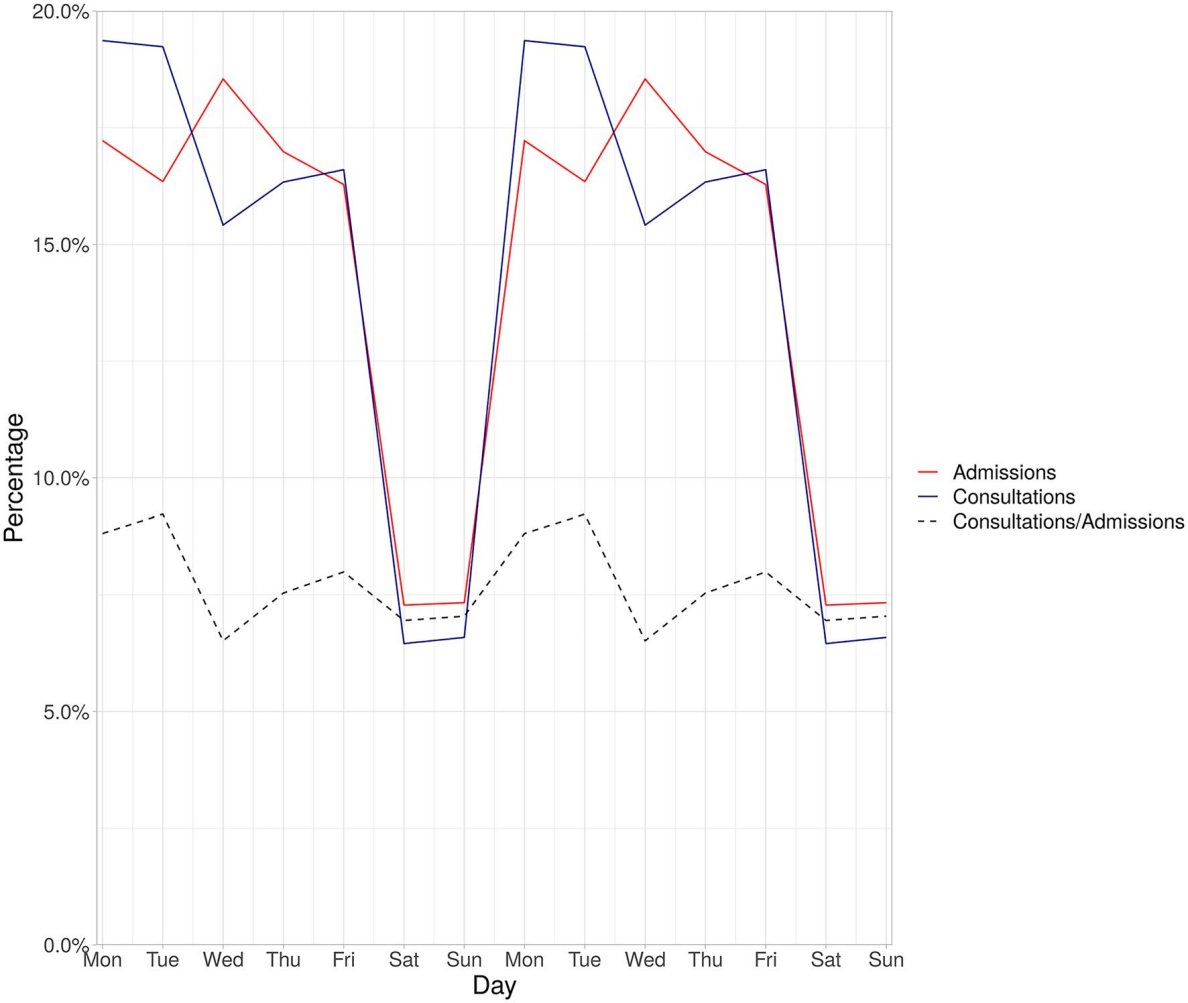
Proportion of overall admissions and consultations stratified per weekday (weekdays duplicated to display two weeks for easier visual perception). The dashed line shows the proportion of patients receiving a consultation among all admissions, stratified per weekday.

In the univariate analysis patients who were admitted during the weekend were much more likely to receive an infection-related consultation during their stay compared to patients admitted during the week (p < 0.001). This effect is however confounded by the other variables in the logistic regression as it was no longer present in the multivariate analysis. Remarkably, an exploratory plot shows that the percentage of admissions and consultations does drop during weekends, reflecting both a lower number of elective admissions and limited consulting capacity. However, the ratio of admissions to consultations remained rather stable (see Fig. 3).

### Predicting need and timing of infection-related consultations

Results for the Fisher’s exact test and Student’s t-test for all baseline characteristics on the train and held-out test set can be found in Supplementary Table 4. No significant differences were found for the baseline characteristics between the train and held-out test set. The developed prediction models reached moderate to high diagnostic accuracy on the cross validation set (mean AUROC range: 0.743–0.890) for the unbalanced dataset depending on the underlying concept, available features, and the applied model technique (Table 3). Model performance did not show any improvements when balancing the data for each of the algorithms for the different modelling concepts. Using the same set of features in the *at-the-door* concept, RF, GBM and LR performed similarly with an AUROC of 0.725, 0.724 and 0.743 respectively. The LR was most suitable for the *at-the-door* concept. Supplementary Fig. 1 shows the distribution of time to consultation at prediction time (i.e. at admission) for the *at-the-door* concept. Furthermore, Supplementary Table 5 shows the LR coefficients for each of the covariates. Model predictions improved using additional time dependent features in the *collapsed* and *time-series* concept compared to the *at-the-door* models (AUROC: 0.861 and 0.890 vs. 0.743). For the *collapsed* and *time-series* concept infection-related consultations were predicted one arbitrary virtual shift ahead (i.e. up to eight hours), with an average of four hours in advance. Supplementary Fig. 2 shows the distribution of time to infection-related consultation at prediction time (i.e. at the start of the arbitrary virtual shift) for the *collapsed* and *time-series* concept. Model performance for collapsed data including the mean, standard deviation, minimum, maximum and trend was similar to collapsed data including only the mean and standard deviation. Therefore, we decided to not include those extra features. For the *collapsed* concept, the RF was the most suitable model (AUROC: 0.861). Supplementary Table 6 shows the model parameters for the best RF model. When removing patients with missing values the performance of the RF and GBM dropped slightly to an AUROC of 0.811, 0.847 and 0.837 for the LR, RF and GBM, respectively. Performance of a LSTM with a time frame of 48 and 80 h with the use of within-eight hours-standard deviation showed no improvements compared to a model without-eight hours-standard deviation. Therefore, the simplest model was used, that is the model without standard deviations of each time dependent feature within all arbitrary virtual shifts. Performance of the LSTM model with a time frame of 48 h improved compared to the *collapsed* concept (AUROC: 0.890 vs. 0.861 for the RF model). However, increasing the time frame for the LSTM to 80 h showed no substantial further improvements (AUROC: 0.890).

**Table 3:** Model performances.

Concept	Algorithm	Patient features (n)	AUROC (train)^b^	Mean AUROC (validation)^b^	95% CI AUROC (validation)^b^	AUROC (test)^b^	Mean AUPRC (validation)^c^	95% CI AUPRC (validation)^c^	AUPRC (test)^c^
At-the-door	RF	9	0.762	0.725	0.707–0.742	0.692	0.160	0.142–0.179	0.125
	GBM	9	0.740	0.724	0.702–0.746	0.704	0.162	0.141–0.183	0.131
	LR	9	0.755	0.743	0.728–0.758	0.724	0.187	0.170–0.204	0.151
Collapsed	RF	104	0.999	0.861	0.849–0.873	0.886	0.334	0.303–0.366	0.447
	GBM	104	1.000	0.855	0.842–0.868	0.887	0.343	0.306–0.379	0.401
	LR^a^	104	0.869	0.811	0.785–0.836	0.841	0.336	0.273–0.400	0.395
Time-series	LSTM (48 h)	104	0.918	0.890	0.875–0.905	0.914	0.449	0.401–0.498	0.541
Time-series	LSTM (80 h)	104	0.920	0.890	0.876–0.904	0.921	0.439	0.383–0.495	0.541

^a^LR was performed on a smaller dataset without missing values (n = 6279).

^b^An AUROC of 0.5 has no discrimination; 0.5–0.7 poor discrimination; 0.7–0.8 acceptable discrimination; 0.8–0.9 excellent discrimination and 0.9 and higher has outstanding discrimination^31^.

^c^Performance is measured by comparing the AUPRC to the baseline (0.078).

**Figure 4:**
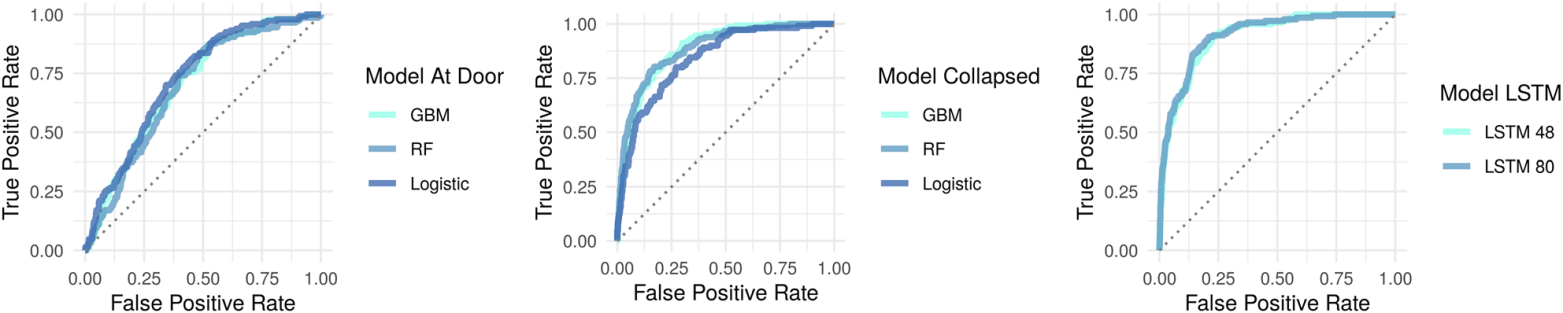
Model performance on the held-out test set by area under the receiver operating curve (AUROC) for each model predicting a consultation at the ICU. LSTM with a time frame of 80 h showed the highest AUROC of 0.921.

Performance on the held-out test set increased with the chosen modelling approach. Compared to the mean performance on the cross validation set the performance on the held-out test set decreased for *at-the-door* models while performance increased for the *collapsed* models as well as for the *time-series* models (48 and 80 h). The AUROC of the LSTM with a time frame of 80 h showed the highest performance on the held-out test set (AUROC: 0.921). Plotting the AUROC revealed the performance of each algorithm per modelling approach on the held-out test set (Fig. 4).

**Figure 5:**
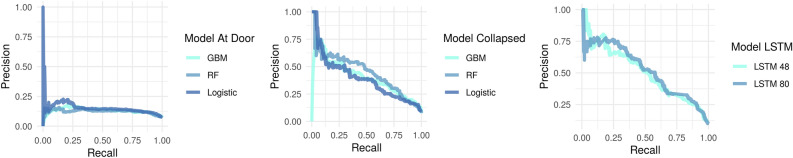
Model performance on the held-out test set by area under the precision recall curve (AUPRC) for each model predicting a consultation at the ICU. The baseline represents the performance of a random classifier and reflects the occurrence of consultations in the study cohort (7.8% of all admissions). LSTM with a time frame of 80 h showed the highest AUPRC of 0.541.

Given the imbalanced dataset of this study, i.e., the majority of admissions (92.2%) did not receive a consultation, assessing the precision recall curve, provides further information on model performance^32^. The precision recall curve shows the trade-off between precision (or positive predictive value) and recall (or sensitivity). Model performance is assessed by comparing the baseline, which reflects the proportion of consultations in the study cohort (0.078), to the AUPRC. A stratified analysis for each algorithm per modelling approach showed that the performance of the models improved with the different modelling approaches (Fig. 5). Again, the performance of the *collapsed* and *time-series* models increased on the held-out test set compared to the mean cross validation results. Consultations were most accurately predicted on the held-out test set by the LSTM with a time frame of 80 h (AUROC: 0.921, AUPRC: 0.541).

### Final architecture of the long short-term memory neural networks

The final architecture of the LSTM model with a time frame of 48 h was similar to the architecture of the LSTM model with a time frame of 80 h. Both models started with a masking layer. This layer was added for patients with a stay shorter than either 48 h or 80 h, depending on the model. The masking layer was followed by a LSTM layer with sixteen units and a drop out of 20%. Depending on the model this layer expected either six time-series steps or ten time series steps (i.e., 48 or 80 h). The final layer was a dense layer with a sigmoid activation function. Twenty epochs and a batch size of 64 led to the most optimal results.

### Calibration, net benefit and model performance for sub-populations

To explore the calibration of the best performing models per modelling approach we evaluated the calibration slope, calibration intercept and the scaled Brier score. The scaled Brier score is computed in a similar way as the Pearson’s R squared, namely the Brier score of the model is compared to the maximum Brier score of a non-informative model^33^. A scaled Brier score close to zero indicates poor calibration while a scaled Brier score of one indicates a perfect calibration. Our analysis revealed that the *at-the-door* model is poorly calibrated (scaled Brier score: 0.033), whereas the calibration of the *collapsed* model is slightly better (scaled Brier score: 0.179). The calibration intercept and slope for the *time-series* concept were close to zero and one, respectively. The scaled Brier score for *time-series* concept were 0.317 and 0.324 for respectively a time frame of 48 and 80 h, which we considered to be a reasonable calibration across individuals. Calibration plots, calibration intercepts, slopes and scaled Brier scores can be found in the Supplementary Fig. 3 and Supplementary Table 7.

Next, we performed a net benefit analysis, which shows how well the model correctly identifies patients in need of an infection-related consultation for different probability thresholds and compares the model against the *treat-all or treat-none strategy*^34^*.* A net benefit analysis for each of the best performing models per modelling approach showed that the *at-the-door* model had no benefit over the *treat-all or treat-none strategy*. On the contrary, the *collapsed* and *time-series* models showed a substantial benefit over the *treat-all* or *treat-none* strategy. The *time-series* models had the highest net benefit across all threshold probabilities (cf. Supplementary Fig. 4). The net benefit of the LSTM with a time frame of 80 h did not show a substantial improvement compared to the LSTM with a time frame of 48 h.

Predictions for different sub-populations of the test set can be found in Supplementary Table 8. For the *at-the-door* concept the AUC for patients with an ICU length of stay (LOS) shorter than two days is considerably higher (0.808). The AUC of readmitted patients, non-post-operative patients, patients with ICU LOS longer than two days and patients who died during their ICU stay is lower (0.568, 0.552, 0.589 and 0.522). For the *collapsed* concept the AUC for the sub-population of patients with a planned admission and post-operative patients is outstanding (0.912 and 0.913). Moreover, the AUC for readmitted patients, non-post-operative patients, patients with an ICU LOS longer than two days and patients who died is lower (0.818, 0.831, 0.800 and 0.740). For the *time-series* concept the AUC for patients with a planned admission and post-operative patients are outstanding for both the 48 and 80 h time frame (0.940 and 0.941, 0.941 and 0.945). The AUC for non-post-operative patients and patients who died was lower (0.862 and 0.828, 0.873 and 0.839). The AUPRC was not directly comparable between different patient groups as it has to be compared to the proportions of patients who received a consultation (base line), which differs per group. However, we included the AUPRC and baseline in Supplementary Table 8. Finally, we explored the performance of the various trained models for different leading times (time from prediction to infection-related consultation). For the *at-the-door* concept we found that overall predictions on the test set were better when the infection-related consultation occurred within the first day after admission compared to later on (AUC: 0.753 vs. 0.686). For the *collapsed* and *time-series* concepts the models did not perform better for patients with shorter vs. longer leading times (see Supplementary Table 9).

### Feature importance

Finally, feature importance plots of the best performing models per modelling approach are presented in Supplementary Fig. 5. The most important variable for the *at-the-door* model using LR was the sending specialty followed by whether the admission was planned or not. For the RF model with the *collapsed* concept the most important feature was SDD, followed by the mean respiratory rate. Feature importance for the LSTM with both time spans were relatively similar. The order for the most important variables were however swapped for the LSTM with a time frame of 48 h and 80 h. The most important variables for the prediction were whether the admission was planned or not and SDD, for the LSTM with a time frame of 48 and 80 h.

## Discussion

This study investigated infection-related consultations (clinical microbiology/ID consultations in this setting) in a cohort of ICU patients and successfully developed a machine learning model for predicting consultations using routine EHR. Globally, ICU patients rarely receive infection-related consultations. Only 7.8% of all admissions in our study cohort received an infection-related consultation creating an imbalanced dataset for the target feature. Our cohort included patients that were admitted to the ICU for monitoring purposes, e.g., post-surgery (64.3% in the no-consultation group). These patients typically stay only for a short amount of time at the ICU if no complications occur (median LOS of 2 days in the no-consultation group). Patients with an infection-related consultation differed significantly from the no-consultation group in age at admission, readmission status, and ICU mortality, thus forming a distinct patient population, this could facilitate better predictions by reducing a “dilution effect”. The explanatory multiple logistic regression model (Table 2) is largely consistent with empiric clinical reasoning and experience, e.g., the low odds for planned admissions to require an infection-related consultation.

Infection-related consultations were predicted in a time shift-based approach up to eight hours in advance (one arbitrary virtual shift). On average consultations were predicted four hours ahead of time. This could support early identification of patients requiring a consultation and support the initiation of subsequent clinical steps (e.g., notifying consultants, performing diagnostics). Performance of the models were measured using AUROC and AUPRC, where an AUROC of 0.5–0.7 is considered poor, 0.7–0.8 acceptable, 0.8–0.9 excellent and 0.9 and higher outstanding^31^ and the AUPRC is compared to the baseline in this study (0.078)^32^. Our incremental approach showed that an *at-the-door* model with baseline patient characteristics is not sufficient. Although achieving a moderate AUROC (0.724), the AUPRC (0.187) of this model was only little above the baseline (0.078). in this imbalanced dataset (7.8% of all patients received a consultation) and was found to be poorly calibrated. Moreover, the best performing *at-the-door* model (LR) cannot handle missing data which frequently occur in EHR data. For demonstration purposes LR was included in the *collapsed* approach with missing data removed. The results demonstrated that RF and GBM models performed better in the *collapsed* approach and include the advantage of being able to handle missing data.

Additional time-dependent features markedly improved model performance. Using a machine learning technique, LSTM, which can work with longitudinal data such as EHR demonstrated strong performance in AUROC and AUPRC measures (AUROC: 0.921, AUPRC: 0.541). This model utilised a dataset comprised with 104 features covering demographic, monitoring, procedural, medication, and diagnostic features. The suitability of LSTM for EHR data was also confirmed in other infection-related studies with similar model performances yet different target events, that is blood culture outcome, sepsis and hospital outcomes such as in hospital mortality and prolonged LOS^25–27^. Although model performances are difficult to compare for different outcomes, our results show strong performance^31^. Interestingly, including more data beyond 48 h (i.e. earlier in the admission) did not substantially improve the LSTM model performance. Possible, non-exclusive explanations for this could be: (i) the large number of patients with a short LOS; and (ii) relevant trigger information might not be detectable in routine EHR data earlier than that. Remarkably, this observation descriptively concurs with routine clinical decision making by many experienced physicians, without necessarily implying a causal relationship.

Analysis of how the different models perform on various sub-populations consistently showed improved performance for non-readmissions, patients with a planned admission, patients admitted via the operation room, patients with a short LOS and patients who survived. These characteristics are often seen in patients who stay in the ICU for post-operative monitoring purposes after elective operations and constitute a substantial part of the patient cohort (e.g.^35^). This patient group, in general, only rarely receives infection-related consultations. The analysis of the feature importance for the best performing models in each modelling approach revealed differences between the models. The *at-the-door* model relied on known features while the *collapsed* model included vital signs (e.g., respiratory rate) that can typically show dynamic changes over time. This could also be observed in the *time-series* approach. However, the feature importance analysis describes solely the functionality of the models. Thus, direct use of these results in clinical decision making, without prior clinical validation is not appropriate. An in-depth clinical interpretation and validation of these results was outside the scope of this proof-of-concept study.

Going beyond explanatory modelling by attempting to predict the occurrence of an infection-related consultation offers several advantages. The diagnosis of infections and thus the need for infection-related consultations, are often highly time-sensitive. In clinical practice a time gain of on average four hours could result in improving allocation and timing of scarce resources, i.e., expert consults, diagnostics, and (timing of) appropriate antimicrobial treatment, which would be beneficial for the patient and all stakeholders involved, intensivists and clinical microbiologists. The positive impact of infection-related consultations was demonstrated in previous studies as mentioned above^2,4–7^. First studies on automating consultations also showed promising findings^9,10^. Despite their reactive nature triggered by the occurrence of clinical events, the results pointed towards the feasibility of data-driven support of consultation workflows. Our proactive approach, i.e., shifting emphasis from reacting to clinical events to predicting an outcome or event, has the potential to bring this to a next level by leveraging existing technology and data. Although no single interventions (e.g., start of antimicrobials) were targeted, and can thus not be directly derived from this approach, this study can serve as a proof-of-principle that clinical action can be predicted. Ultimately, sepsis would be one of the most prominent examples often requiring immediate action based on the mere suspicion of sepsis^1^. Initiating diagnostic procedures and adequate treatment, antimicrobial therapy in particular, and the correct and timely ordering of those are essential for improving quality of care for critically ill patients. Rapid diagnostics in the (microbiological) laboratory or at point-of-care have evolved greatly over recent years and have the potential to reduce turnaround time substantially, i.e. time from order to clinical action^36,37^. However, technical solutions in the laboratory can be costly and do not solve the problem of optimal resource allocation across ICU patients. Potential pre-analytical time gains are often less considered when discussing the concept of rapid diagnostics, but the vital aspects of the pre-analytical time and workflow are well established^38^. These considerations also apply to clinical workflows of physicians working in multi-disciplinary teams at the ICU, including infection-related consultations. Moreover, our study did not include microbiological data beyond the consultation time stamps. Infection-related consultations were rather predicted using only routine ICU data. Thus, one useful case scenario of this prediction model could also be the support of (timely) initiation of microbiological diagnostics and appropriate antimicrobial therapy.

There is a number of issues that need to be considered carefully: Firstly, since some features in our study were relatively sparse, aggregation of data into arbitrary virtual 8-h shifts was necessary. Secondly, since we have used “stored” data for this proof-of-concept study, the developed models need to be trained with more recent data prior to clinical validation and implementation. Thirdly, both training and test datasets were used from a single institution and external validation is needed. The patient population and care might be different in other institutions. For instance, other institutions might have different infrastructure, logistics, level of care, or patient populations. This can impact the generalisability of our trained model. Therefore, before clinical use in other institutions, the model potentially has to be retrained and validated with appropriate data. In addition, since SDD is a standard procedure in our hospital it is plausible that this has been identified as a variable of importance in the *collapsed* and *time-series* approach. SDD might identify the non-elective sub cohort and proved a “cleaner” medical background (e.g., reduce the probability of early infections). Next, although we tested our model against a held-out dataset, validation and usability in a real-time scenario need yet to be demonstrated in clinical practice. Moreover, in clinical practice, predictions by the *collapsed* and *time-series* approach have to be made using a rolling window prediction system, where the input data is continuously updated by adding new observations every eight hours in the current setup. To provide further clarity, after each eight hour shift, we utilise a partly new 48-h subset of the data, and our model will predict for the subsequent shift. Our approach is in agreement with other studies (albeit with other clinical outcomes)^39,40^ and the envisioned implementation in clinical practice is consistent with the way our model was trained. However, this approach implicitly assumes that the time series dynamics signalling "consultation vs. no consultation" remain relatively consistent throughout a patient's stay in the ICU at different time points. While this is a plausible assumption, it could affect the performance of the model in clinical practice. Finally, we worked with data that reflect human decisions (clinical procedures as features, infection-related consultation as target outcome). In practice this could influence the model performance as human decisions might change, based on these respective predictions. Of note: machine learning models are known to degrade in performance once deployed. Thus, optimal maintenance is always important^41^.

We would like to mention several strengths of this study: Firstly, all features used in the final model are generic features routinely available at ICUs (see Supplementary Table 2). This can facilitate external validation and potential implementation in local EHR systems. Secondly, the models have a good to  strong overall performance despite using arbitrary virtual shifts and the time stamp for registration of consultations, which do not necessarily match the timing of actual clinical decision making. This partial delay was due to using the available time stamp of registration of consults, rather than of the actual consultations. Thirdly, surprisingly, prediction of infection-related consultations worked well, even without using a main standard trigger, i.e. diagnostic results from medical microbiology. Including this information might further increase the already strong performance of the presented models. Finally, we consider workflow-centred approaches (e.g., the one presented here) an easier accessible way to familiarise medical staff with sophisticated machine learning concepts in contrast to more implication-heavy models (e.g., sepsis prediction). Although LSTM models do not offer straightforward and interpretable measures such as, for example, partial dependence, or individual conditional expectation, this could substantially lower the barriers for deployment of machine learning to clinical use and acceptance. Therefore, we conclude that machine learning-supported approaches, such as the one presented here, have great potential to further improve patient care and clinical outcome for critically ill patients with (suspected) infections.

### Supplementary Information

Supplementary Information.

## Data Availability

The data is registered in the Groningen Data Catalogue (https://groningendatacatalogus.nl). The data used in this study are not publicly available. These data are, however, available from the corresponding author (LRZ) upon reasonable request and with compliance to the General Data Protection Regulation (GDPR) in the EU. Access to computer code used in this study is available upon request to the corresponding author.
