# Supplementary material for: Identifying the need for infection-related consultations in intensive care patients using machine learning models
Source: Sci Rep. 2024 Jan 28;14:2317. doi: 10.1038/s41598-024-52741-w (PMC10822855; doi:10.1038/s41598-024-52741-w)

**Supplementary files**

**Identifying the need for infection-related consultations in intensive care patients using machine learning models**

Leslie R. Zwerwer^1,2*†^, Christian F. Luz^3*^, Dimitrios Soudis^2^, Nicoletta Giudice^2^, Maarten W. N. Nijsten^4^, Corinna Glasner^3^, Maurits H. Renes^4^, Bhanu Sinha^3^

1 University of Groningen, University Medical Center Groningen, Department of Health Sciences, Hanzeplein 1, 9713 GZ Groningen, The Netherlands

2 University of Groningen, Center for Information Technology, Nettelbosje 1, 9747 AJ Groningen, The Netherlands

3 University of Groningen, University Medical Center Groningen, Department of Medical Microbiology and Infection Prevention, Hanzeplein 1, 9713 GZ Groningen, The Netherlands

4 University of Groningen, University Medical Center Groningen, Department of Critical Care, Hanzeplein 1, 9713 GZ Groningen, The Netherlands

* Equal contribution

^†^ Corresponding author

**Supplementary Table 1.** Missingness for the full data set (per feature) after eight-hours aggregation.

**Supplementary Table 2.** Features used per concept.

**Supplementary Table 3.** Tripod checklist template.

**Supplementary Table 4.** Patient characteristics stratified by train and held-out test set.

**Supplementary Table 5.** Model coefficients for best performing at-the-door model (i.e., logistic regression)**.**

**Supplemental Table 6.** Optimal random forest parameters (i.e., best performing model collapsed concept).

**Supplementary Table 7.** Calibration measures for best performing model per modelling concept.

**Supplementary Table 8.** Performance of the best performing model per modelling concept for sub-populations of the test set.

**Supplementary Table 9**. Model performance for different leading times.

**Supplementary Figure 1.** Histogram of time to infection-related consultation at prediction time for the at-the-door concept (i.e., at ICU admission).

**Supplementary Figure 2.** Histogram of time to infection-related consultation at prediction time for the collapsed and time-series concept.

**Supplementary Figure 3.** Calibration plots of the best performing models per modelling approach.

**Supplementary Figure 4.** Net benefit analysis of the best performing models per modelling approach compared to a treat all and treat none strategy.

**Supplementary Figure 5.** Feature importance for best performing models per modelling approach.

**Supplementary Table 1.** *Missingness for the full data set (per feature) after eight-hours aggregation.* Of note, the laboratory and point of care tests, prescriptions and line placements features have two types of missing data, data that is truly missing and data that is missing because there was no event. The percentage missing is calculated as: $\frac{Total number of missing events}{Total number of virtual shifts for all patients}*100$.

| Feature | Percentage missing |
| --- | --- |
| Admission sending specialty | 0.00 |
| Weekend admission | 0.00 |
| Age at admission | 0.00 |
| Body mass index | 0.00 |
| Gender | 0.00 |
| Mechanical ventilation at admission | 0.00 |
| Planned admission | 0.00 |
| Readmission | 0.00 |
| Admission via operation room | 0.00 |
| Antimicrobials (IV) | 1.62 |
| Antimicrobials (other) | 1.62 |
| Antimicrobials (oral) | 1.62 |
| Arterial line | 1.47 |
| Central venous catheter | 1.47 |
| Diuretics (IV) | 1.62 |
| Diuretics (other) | 1.62 |
| Diuretics (oral) | 1.62 |
| Crystalloids (infusion) | 1.62 |
| Crystalloids (IV) | 1.62 |
| Crystalloids (other) | 1.62 |
| Culture (blood) | 95.51 |
| Culture (line tip) | 99.17 |
| Culture (throat) | 91.93 |
| Culture (liquor) | 98.15 |
| Culture (nose, throat, perineum) | 97.49 |
| Culture (nose, perineum) | 98.54 |
| Culture (rectum) | 91.46 |
| Culture (sputum) | 93.72 |
| Culture (urine) | 96.50 |
| Dialysis catheter | 1.47 |
| Duodenal tube | 1.47 |
| Haemostatic agents (oral or subcutaneous) | 1.62 |
| Inhalative agents | 1.62 |
| Gastric tube (14ch) | 1.47 |
| Gastric tube (16ch) | 1.47 |
| Pacemaker wires | 1.47 |
| Peripheral line (14g) | 1.47 |
| Peripheral line (16g) | 1.47 |
| Peripheral line (18g) | 1.47 |
| Peripheral line (20g) | 1.47 |
| Peripheral line (22g) | 1.47 |
| Selective digestive decontamination | 1.62 |
| Sedatives/analgesics (IV) | 1.62 |
| Sedatives/analgesics (other) | 1.62 |
| Sedatives/analgesics (oral) | 1.62 |
| Arterial blood pressure (diastolic) | 8.81 |
| Arterial blood pressure (mean) | 9.00 |
| Arterial blood pressure (systolic) | 8.97 |
| Catheter (14fr) | 1.47 |
| Catheter (16fr) | 1.47 |
| FiO_2_ | 55.03 |
| Glasgow coma scale | 34.21 |
| Heart rate | 1.29 |
| Pulse rate | 2.67 |
| Albumine (urine) 24-hour mg | 94.25 |
| Albumine (urine) mg/l | 81.19 |
| Alkaline phosphatase (blood) U/l | 71.41 |
| Amylase (blood) | 71.95 |
| Bilirubin direct (blood) µmol/l | 96.89 |
| Bilirubin total (blood) µmol/l | 96.93 |
| Calcium (blood) mmol/l | 71.58 |
| Chloride (blood) mmol/l | 59.79 |
| Creatinine kinase (blood) U/l | 54.64 |
| Bilirubin direct (blood; additional measure) µmol/l | 89.04 |
| Protein total (blood) g/l | 82.79 |
| Erythroblasts (blood) 10^9^/l | 99.20 |
| Fibrinogen (blood) g/l | 83.46 |
| Phosphate (blood) mmol/l | 59.70 |
| Gamma-glutamyl transferase (blood) U/L | 71.43 |
| Glucose (blood) mmol/l | 97.79 |
| Haemoglobin (blood) mmol/l | 57.40 |
| Haematocrit (blood) | 58.99 |
| International normalized ratio (blood) | 96.04 |
| Potassium 24-hour (urine) mmol | 90.28 |
| Potassium (blood) mmol/l | 97.10 |
| Creatinine 24 hours (urine) mmol | 96.67 |
| Creatinine (blood) µmol/l | 59.04 |
| Lactate dehydrogenase (blood) U/l | 59.22 |
| Leucocytes (blood) 10^9^/l | 59.03 |
| Magnesium (blood) mmol/l | 59.56 |
| Mean corpuscular volume (blood) fl | 84.71 |
| Sodium 24 hours (urine) mmol | 89.92 |
| Sodium (blood) mmol/l | 59.04 |
| Base excess (point of care) mmol/l | 14.54 |
| Arterial HCO_3_ (point of care) mmol/l | 14.40 |
| Arterial pCO_2_ (point of care) kPa | 14.17 |
| Arterial pH | 14.20 |
| Arterial oxygen saturation | 14.16 |
| Calcium ionized (point of care) mmol/l | 64.16 |
| Chloride (point of care) mmol/l | 69.43 |
| Glucose (point of care) mmol/l | 12.60 |
| Haemoglobin (point of care) mmol/l | 12.60 |
| Methaemoglobin (point of care) % | 59.44 |
| Potassium (point of care) mmol/l | 12.53 |
| Lactate (point of care) mmol/l | 13.55 |
| Sodium (point of care) mmol/l | 12.61 |
| Bilirubin total (blood; additional measure) µmol/l | 89.03 |
| Protein total (blood; additional measure) g/l | 91.43 |
| Thrombocytes (blood) 10^9/^l | 54.84 |
| Troponine T (blood) ng/l | 65.92 |
| Urea 24 hours (urine) mmol | 91.19 |
| Urea (blood) mmol/l | 59.10 |
| Respiration rate | 3.23 |
| Temperature | 50.41 |

Point of care tests were also performed on blood.

| **Supplementary Table 2.** *Features used per concept*. |  |  |  |
| --- | --- | --- | --- |
| Feature |  | Concept |  |
|  | *At-the-door* | *Collapsed* | *Time-series* |
| Admission sending specialty | X | X | X |
| Weekend admission | X | X | X |
| Age at admission | X | X | X |
| Body mass index | X | X | X |
| Gender | X | X | X |
| Mechanical ventilation at admission | X | X | X |
| Planned admission | X | X | X |
| Readmission | X | X | X |
| Admission via operation room | X | X | X |
| Antimicrobials (IV) | | X | X |
| Antimicrobials (other) | | X | X |
| Antimicrobials (oral) | | X | X |
| Arterial line | | X | X |
| Central venous catheter | | X | X |
| Diuretics (IV) | | X | X |
| Diuretics (other) | | X | X |
| Diuretics (oral) | | X | X |
| Crystalloids (infusion) | | X | X |
| Crystalloids (IV) | | X | X |
| Crystalloids (other) | | X | X |
| Culture (blood) | | X | X |
| Culture (line tip) | | X | X |
| Culture (throat) | | X | X |
| Culture (liquor) | | X | X |
| Culture (nose, throat, perineum) | | X | X |
| Culture (nose, perineum) | | X | X |
| Culture (rectum) | | X | X |
| Culture (sputum) | | X | X |
| Culture (urine) | | X | X |
| Dialysis catheter | | X | X |
| Duodenal tube | | X | X |
| Haemostatic agents (oral or subcutaneous) | | X | X |
| Inhalative agents | | X | X |
| Gastric tube (14ch) | | X | X |
| Gastric tube (16ch) | | X | X |
| Pacemaker wires | | X | X |
| Peripheral line (14g) | | X | X |
| Peripheral line (16g) | | X | X |
| Peripheral line (18g) | | X | X |
| Peripheral line (20g) | | X | X |
| Peripheral line (22g) | | X | X |
| Selective digestive decontamination |  | X | X |
| Sedatives/analgesics (IV) | | X | X |
| Sedatives/analgesics (other) | | X | X |
| Sedatives/analgesics (oral) | | X | X |
| Arterial blood pressure (diastolic) |  | X | X |
| Arterial blood pressure (mean) |  | X | X |
| Arterial blood pressure (systolic) |  | X | X |
| Catheter (14fr) | | X | X |
| Catheter (16fr) | | X | X |
| FiO2 |  | X | X |
| Glasgow coma scale | | X | X |
| Heart rate |  | X | X |
| Pulse rate |  | X | X |
| Albumine (urine) 24 hours mg * |  | X | X |
| Albumine (urine) mg/l * |  | X | X |
| Alkaline phosphatase (blood) U/l * |  | X | X |
| Amylase (blood) * |  | X | X |
| Bilirubin direct (blood) µmol/l * |  | X | X |
| Bilirubin total (blood) µmol/l * |  | X | X |
| Calcium (blood) mmol/l * |  | X | X |
| Chloride (blood) mmol/l * |  | X | X |
| Creatinine kinase (blood) U/l * | | X | X |
| Bilirubin direct (blood; additional measure) µmol/l * |  | X | X |
| Protein total (blood) g/l * |  | X | X |
| Erythroblasts (blood) 10^9^/l |  | X | X |
| Fibrinogen (blood) g/l * |  | X | X |
| Phosphate (blood) mmol/l * |  | X | X |
| Gamma-glutamyl transferase (blood) u/l * |  | X | X |
| Glucose (blood) mmol/l * |  | X | X |
| Haemoglobin (blood) mmol/l * |  | X | X |
| Haematocrit (blood) * | | X | X |
| International normalized ratio (blood) * | | X | X |
| Potassium 24-hour (urine) mmol * |  | X | X |
| Potassium (blood) mmol/l * |  | X | X |
| Creatinine 24 hours (urine) mmol * |  | X | X |
| Creatinine (blood) µmol/l * |  | X | X |
| Lactate dehydrogenase (blood) U/l * | | X | X |
| Leucocytes (blood) 10^9^/l * |  | X | X |
| Magnesium (blood) mmol/l * |  | X | X |
| Mean corpuscular volume (blood) fl * | | X | X |
| Sodium 24 hours (urine) mmol * |  | X | X |
| Sodium (blood) mmol/l * |  | X | X |
| Base excess (point of care) mmol/l * |  | X | X |
| Arterial HCO_3_ (point of care) mmol/l * |  | X | X |
| Arterial pCO_2_ (point of care) kPa * |  | X | X |
| Arterial pH * |  | X | X |
| Arterial oxygen saturation * |  | X | X |
| Calcium ionized (point of care) mmol/l * |  | X | X |
| Chloride (point of care) mmol/l * |  | X | X |
| Glucose (point of care) mmol/l * |  | X | X |
| Haemoglobin (point of care) mmol/l * |  | X | X |
| Methaemoglobin (point of care) % * |  | X | X |
| Potassium (point of care) mmol/l * |  | X | X |
| Lactate (point of care) mmol/l * |  | X | X |
| Sodium (point of care) mmol/l * |  | X | X |
| Bilirubin total (blood; additional measure) µmol/l * |  | X | X |
| Protein total (blood; additional measure) g/l * |  | X | X |
| Thrombocytes (blood) 10^9/^l * |  | X | X |
| Troponine T (blood) ng/l * |  | X | X |
| Urea 24 hours (urine) mmol * |  | X | X |
| Urea (blood) mmol/l * |  | X | X |
| Respiration rate | | X | X |
| Temperature | | X | X |
| * low/normal/high according to reference range. Point of care tests were also performed on blood. | |  |  |

| **Supplementary Table 3.** *Tripod checklist template^30^* | | | |
| --- | --- | --- | --- |
| **Section/Topic** | **Item** | **Checklist Item** | **Page** |
| **Title and abstract** | | | |
| Title | 1 | Identify the study as developing and/or validating a multivariable prediction model, the target population, and the outcome to be predicted. | 1 |
| Abstract | 2 | Provide a summary of objectives, study design, setting, participants, sample size, predictors, outcome, statistical analysis, results, and conclusions. | 2 |
| **Introduction** | | | |
| Background and objectives | 3a | Explain the medical context (including whether diagnostic or prognostic) and rationale for developing or validating the multivariable prediction model, including references to existing models. | 3-4 |
|  | 3b | Specify the objectives, including whether the study describes the development or validation of the model or both. | 4 |
| **Methods** | | | |
| Source of data | 4a | Describe the study design or source of data (e.g., randomized trial, cohort, or registry data), separately for the development and validation data sets, if applicable. | 4-6 |
|  | 4b | Specify the key study dates, including start of accrual; end of accrual; and, if applicable, end of follow-up. | 4 |
| Participants | 5a | Specify key elements of the study setting (e.g., primary care, secondary care, general population) including number and location of centres. | 4 |
|  | 5b | Describe eligibility criteria for participants. | 4 |
|  | 5c | Give details of treatments received, if relevant. | 4-6, supplementary files Table 2 |
| Outcome | 6a | Clearly define the outcome that is predicted by the prediction model, including how and when assessed. | 5-8 |
|  | 6b | Report any actions to blind assessment of the outcome to be predicted. | - |
| Predictors | 7a | Clearly define all predictors used in developing or validating the multivariable prediction model, including how and when they were measured. | 4-8 Supplementary Table 2 |
|  | 7b | Report any actions to blind assessment of predictors for the outcome and other predictors. | - |
| Sample size | 8 | Explain how the study size was arrived at. | 4 |
| Missing data | 9 | Describe how missing data were handled (e.g., complete-case analysis, single imputation, multiple imputation) with details of any imputation method. | 5-6 |
| Statistical analysis methods | 10a | Describe how predictors were handled in the analyses. | 7-8 |
|  | 10b | Specify type of model, all model-building procedures (including any predictor selection), and method for internal validation. | 7-9 |
|  | 10d | Specify all measures used to assess model performance and, if relevant, to compare multiple models. | 8 |
| Risk groups | 11 | Provide details on how risk groups were created, if done. | - |
| **Results** | | | |
| Participants | 13a | Describe the flow of participants through the study, including the number of participants with and without the outcome and, if applicable, a summary of the follow-up time. A diagram may be helpful. | 9-10 |
|  | 13b | Describe the characteristics of the participants (basic demographics, clinical features, available predictors), including the number of participants with missing data for predictors and outcome. | 9-10 |
| Model development | 14a | Specify the number of participants and outcome events in each analysis. | 9-10,  Supplementary Table 4 |
|  | 14b | If done, report the unadjusted association between each candidate predictor and outcome. | 10-11 |
| Model specification | 15a | Present the full prediction model to allow predictions for individuals (i.e., all regression coefficients, and model intercept or baseline survival at a given time point). | 10-11 14-15 Supplementary Tables 5 and 6 |
|  | 15b | Explain how to the use the prediction model. | 18 |
| Model performance | 16 | Report performance measures (with Cis) for the prediction model. | 13 |
| **Discussion** | | | |
| Limitations | 18 | Discuss any limitations of the study (such as nonrepresentative sample, few events per predictor, missing data). | 17-18 |
| Interpretation | 19b | Give an overall interpretation of the results, considering objectives, limitations, and results from similar studies, and other relevant evidence. | 16-17 |
| Implications | 20 | Discuss the potential clinical use of the model and implications for future research. | 17 |
| **Other information** | | | |
| Supplementary information | 21 | Provide information about the availability of supplementary resources, such as study protocol, Web calculator, and data sets. | 19 |
| Funding | 22 | Give the source of funding and the role of the funders for the present study. | 19 |

| **Supplementary Table 4.** *Patient characteristics stratified by train and held-out test set.* | | | |
| --- | --- | --- | --- |
|  | Train (N=7747) | Test (N=1937) | p-value* |
| Consultation |  |  | 0.671 |
| Yes | 612 (7.9%) | 147 (7.6%) |  |
| No | 7,135 (92.1%) | 1,790 (92.4%) |  |
| Gender |  |  | 0.896 |
| Female | 2,998 (38.7%) | 753 (38.9%) |  |
| Male | 4,749 (61.3%) | 1,184 (61.1%) |  |
| Age at admission |  |  | 0.773 |
| Mean (SD) | 60.4 (15.2) | 60.3 (15.4) |  |
| Range | 18 - 101 | 18 - 95 |  |
| Readmission |  |  | 0.8517 |
| No | 7,123 (91.9%) | 1,784 (92.1%) |  |
| Yes | 624 (8.1%) | 153 (7.9%) |  |
| Admission via operation room |  |  | 0.4175 |
| Not via OR | 2,937 (37.9%) | 754 (38.9%) |  |
| Via OR | 4,810 (62.1%) | 1,183 (61.1%) |  |
| Planned admission | | | 0.3343 |
| No | 4,003 (51.7%) | 977 (50.4%) |  |
| Yes | 3,744 (48.3%) | 960 (49.6%) |  |
| Length of ICU stay (days) |  |  | 0.8271 |
| Mean (SD) | 4.0 (6.2) | 4.0 (6.1) |  |
| Median | 2 | 2 |  |
| Range | 1 - 119 | 1 - 71 |  |
| Hospital mortality (ICU) |  |  | 0.3306 |
| Died | 581 (7.5%) | 132 (6.8%) |  |
| Survived | 7,166 (92.5%) | 1,805 (93.2%) |  |
| *) Comparing train vs. held-out test set; Fisher’s exact test for categorical features; Student’s t-test for continuous features. | | | |

**Supplementary Table 5.** *Model coefficients for best performing at-the-door model (i.e., logistic regression)***.**

| Feature |  | Coefficient | Odds ratio |
| --- | --- | --- | --- |
| Gender |  |  |  |
|  | Female | 0.00 | 1.00 |
|  | Male | -0.01 | 0.99 |
| Age at admission |  | 0.01 | 1.01 |
| Mechanical ventilation at admission |  |  |  |
|  | No | 0.00 | 1.00 |
|  | Yes | 0.62 | 1.85 |
| Planned admission |  |  |  |
|  | No | 0.00 | 1.00 |
|  | Yes | -1.26 | 0.28 |
| Number of readmissions |  | 0.3 | 1.35 |
| Admitted via operation room |  |  |  |
|  | No | 0.00 | 1.00 |
|  | Yes | -0.43 | 0.65 |
| Body mass index |  | 0.01 | 1.02 |
| Admission during the weekend |  | -0.11 | 0.89 |
| Admission sending specialty |  |  |  |
|  | Allergology | 0.00 | 1.00 |
|  | Anesthesiology | 0.49 | 1.63 |
|  | Cardio-pulmonary surgery | -0.74 | 0.48 |
|  | Cardiology | 0.01 | 1.01 |
|  | Dermatology | -0.06 | 0.94 |
|  | Gastroenterology | 0.09 | 1.1 |
|  | Gynaecology | -0.27 | 0.76 |
|  | Surgery | 0.27 | 1.31 |
|  | Haematology | 1.36 | 3.9 |
|  | Hepatology | -0.25 | 0.78 |
|  | Internal medicine | 0.4 | 1.49 |
|  | Throat, nose, ear surgery | -0.07 | 0.94 |
|  | Lung diseases | 0.46 | 1.59 |
|  | Oral diseases and jaw surgery | -0.76 | 0.47 |
|  | Nephrology | -0.11 | 0.89 |
|  | Neuro surgery | -0.05 | 0.95 |
|  | Neurology | -0.05 | 0.95 |
|  | Oncology | 0.56 | 1.75 |
|  | Ophthalmology | -0.07 | 0.93 |
|  | Orthopaedics | -0.41 | 0.66 |
|  | Plastic surgery | -0.37 | 0.69 |
|  | Psychiatry | -1.14 | 0.32 |
|  | Radiology | 0.00 | 1.00 |
|  | Radiotherapy | -0.07 | 0.93 |
|  | Rheumatologist | 0.42 | 1.52 |
|  | Rehabilitation | -0.02 | 0.98 |
|  | Thoracic surgery | -0.02 | 0.98 |
|  | Traumatology | 0.00 | 1.00 |
|  | Urology | -0.12 | 0.88 |
|  | Vascular surgery | -0.01 | 0.99 |

**Supplemental Table 6.** *Optimal random forest parameters (i.e., best performing model collapsed concept).*

| **Parameter** | **Value** |
| --- | --- |
| Number of features to sample at each split (mtries) | 26 |
| Minimum number of observations in a leaf to split (min_rows) | 4 |
| Maximum tree depth (max_depth) | 45 |
| Sample rate (sample_rate) | 0,7 |
| Number of trees (ntrees) | 428 |

| **Supplementary Table 7.** *Calibration measures for best performing model per modelling concept.* | | | | |
| --- | --- | --- | --- | --- |
|  | Model | | | |
|  | *At-the-door*  Logistic regression | *Collapsed*  Random forest | *Time-series*  LSTM48 | LSTM80 |
| Intercept | -0.054 | -0.070 | -0.005 | -0.001 |
| Slope | 0.813 | 1.378 | 1.077 | 1.096 |
| Scaled Brier score | 0.033 | 0.179 | 0.317 | 0.324 |

**Supplementary Table 8.** *Performance of the best performing model per modelling concept for sub-populations of the test set.*

|  | *At-the-door* concept | | *Collapsed* concept | | *Time-series* concept  (48 hours) | | | *Time-series* concept  (80 hours) | | All concepts | All concepts |
| --- | --- | --- | --- | --- | --- | --- | --- | --- | --- | --- | --- |
|  | AUC | AUPRC | AUC | AUPRC | AUC | AUPRC | | AUC | AUPRC | Proportion of consultations (base line) | Number of admissions |
| Age* |  |  |  |  |  |  | |  |  |  |  |
| < 63 years | 0.680 | 0.116 | 0.883 | 0.524 | 0.906 | 0.564 | | 0.913 | 0.564 | 0.076 | 936 |
| ≥ 63 years | 0.764 | 0.194 | 0.887 | 0.392 | 0.922 | 0.531 | | 0.927 | 0.524 | 0.076 | 1,001 |
| Readmission |  |  |  |  |  | |  |  |  |  |  |
| No | 0.732 | 0.166 | 0.892 | 0.443 | 0.917 | 0.516 | | 0.922 | 0.517 | 0.071 | 1,784 |
| Yes | 0.568 | 0.138 | 0.818 | 0.519 | 0.883 | 0.713 | | 0.910 | 0.754 | 0.131 | 153 |
| Admission via ooperation room |  |  |  |  |  |  | |  |  |  |  |
| No | 0.552 | 0.162 | 0.831 | 0.506 | 0.862 | 0.554 | | 0.873 | 0.573 | 0.134 | 754 |
| Yes | 0.763 | 0.118 | 0.913 | 0.315 | 0.941 | 0.513 | | 0.945 | 0.486 | 0.039 | 1,183 |
| Planned admission |  |  |  |  |  |  | |  |  |  |  |
| No | 0.615 | 0.161 | 0.850 | 0.471 | 0.881 | 0.568 | | 0.893 | 0.568 | 0.116 | 977 |
| Yes | 0.764 | 0.166 | 0.912 | 0.391 | 0.940 | 0.487 | | 0.941 | 0.486 | 0.035 | 960 |
| ICU length of stay* |  |  |  |  |  |  | |  |  |  |  |
| ≤ 2 days | 0.808 | 0.057 | 0.898 | 0.330 | 0.905 | 0.211 | | 0.908 | 0.215 | 0.019 | 1,224 |
| > 2 days | 0.589 | 0.218 | 0.800 | 0.516 | 0.876 | 0.652 | | 0.885 | 0.666 | 0.174 | 713 |
| Mortality |  |  |  |  |  |  | |  |  |  |  |
| No | 0.682 | 0.081 | 0.850 | 0.256 | 0.894 | 0.372 | | 0.900 | 0.350 | 0.045 | 1,805 |
| Yes | 0.522 | 0.530 | 0.740 | 0.707 | 0.828 | 0.829 | | 0.839 | 0.828 | 0.492 | 132 |

* Grouped by median (rounded to whole numbers).

**Supplementary Table 9**. *Model performance for different leading times.* Model performance for different leading times, that is time from prediction to infection-related consultation (at admission for *at-the-door* concept and at the beginning of the virtual shift for the *collapsed* and *time-series* concept).

|  | *At-the-door* concept | |  |  | *Collapsed* concept | | *Time-series* concept  (48 hours) | | *Time-series* concept  (80 hours) | | *Collapsed* and *time-series* concept | |
| --- | --- | --- | --- | --- | --- | --- | --- | --- | --- | --- | --- | --- |
|  | AUC | AUPRC | Proportion of consultations  (base line) | Number of admissions | AUC | AUPRC | AUC | AUPRC | AUC | AUPRC | Proportion of consultations (base line) | Number of admissions |
| Leading times* |  |  |  |  |  |  |  |  |  |  |  |  |
| Group 1 | 0.753 | 0.110 | 0.044 | 1873 | 0.875 | 0.255 | 0.895 | 0.397 | 0.904 | 0.386 | 0.038 | 1860 |
| Group 2 | 0.686 | 0.059 | 0.035 | 1854 | 0.896 | 0.351 | 0.932 | 0.426 | 0.936 | 0.423 | 0.041 | 1867 |

*Grouped by the median leading time (rounded to whole number), that is, respectively, less and more than one day for the *at-the-door* concept and less and more than four hours for the *collapsed* and *time-series* concept.

**Supplementary Figure 1.** *Histogram of time to infection-related consultation at prediction time for the at-the-door concept (i.e., at ICU admission).*


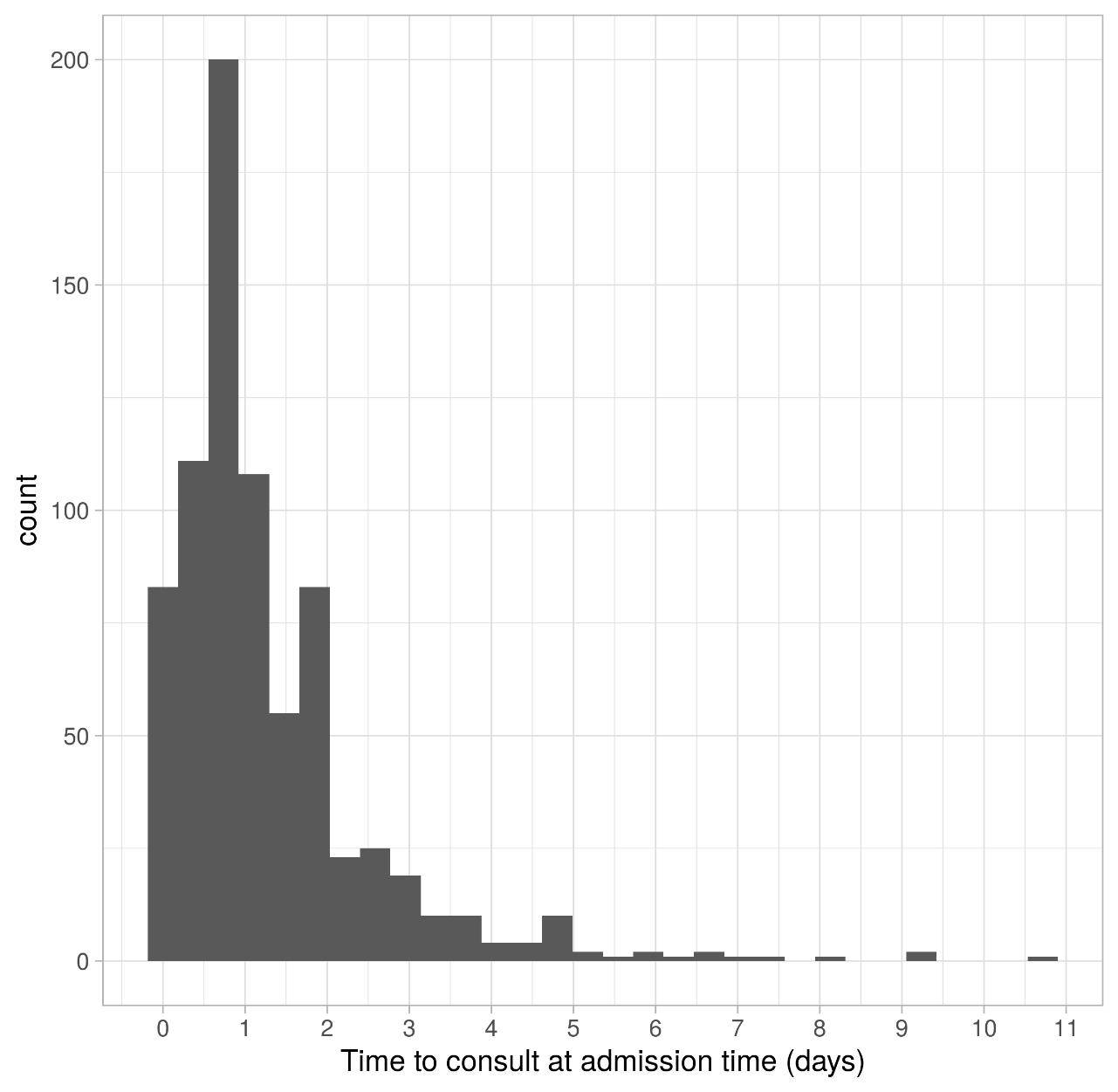


**Supplementary Figure 2.** *Histogram of time to infection-related consultation at prediction time for the collapsed and time-series concept.* ***
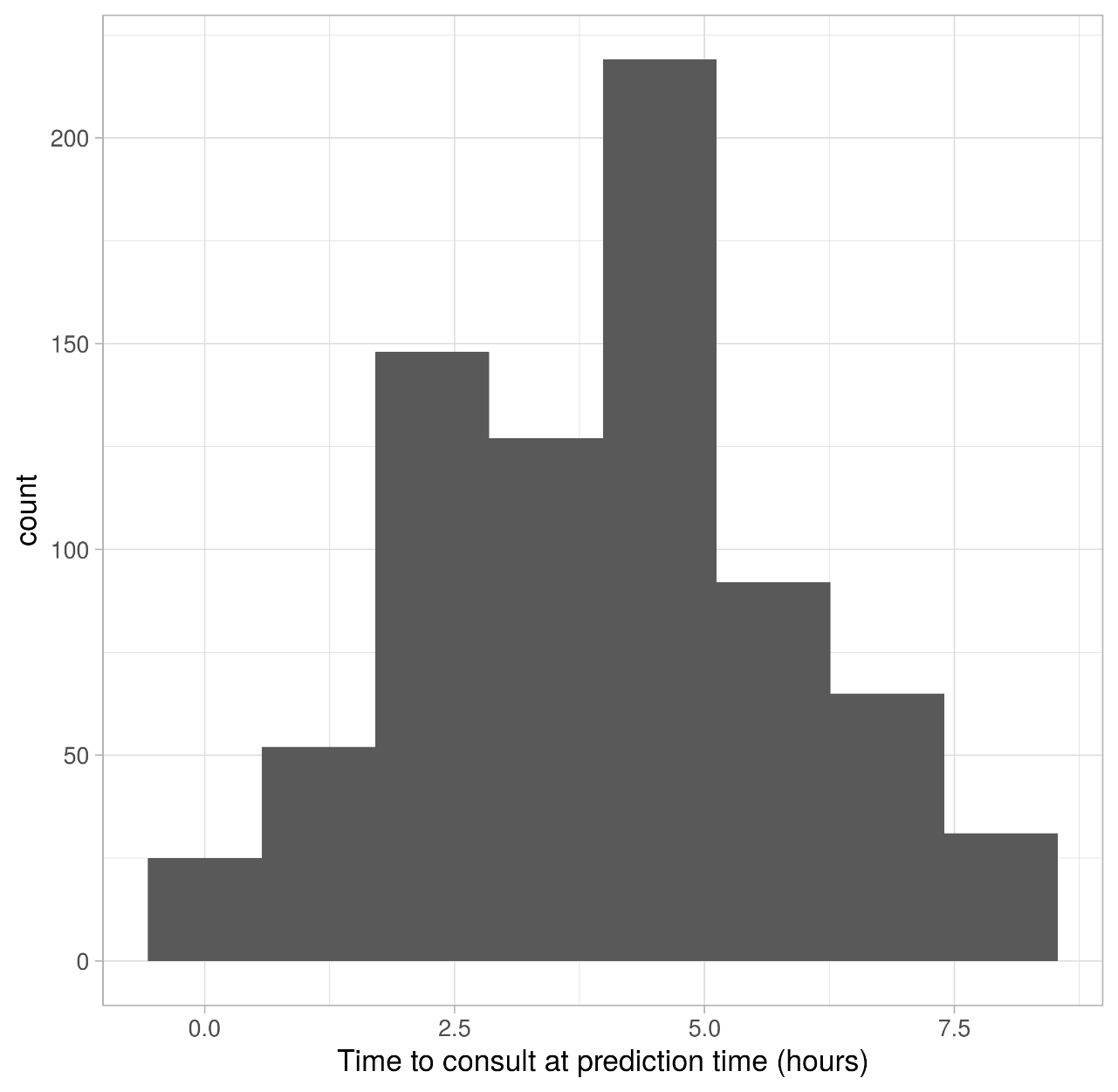
***

**Supplementary Figure 3.** *Calibration plots of the best performing models per modelling approach.* A) Calibration of *at-door* logistic regression model, B) calibration of random forest model for the *collapsed* approach, C) calibration of long short-term memory neural network with a time frame of 48 hours, D) calibration of long short-term memory neural network with a time frame of 80 hours.

| **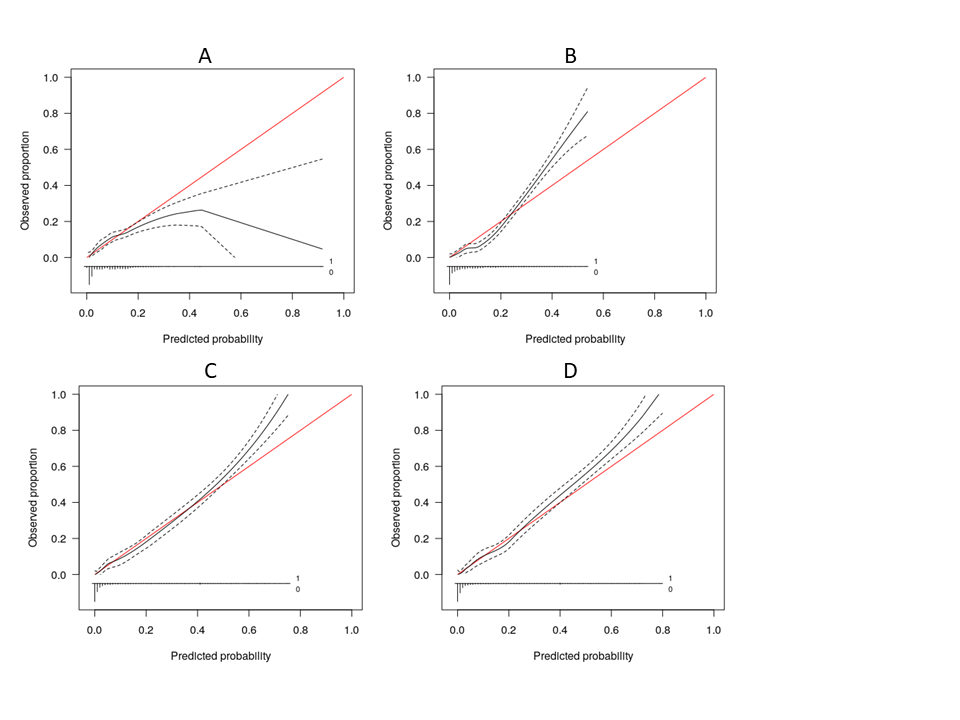** |
| --- |

**Supplementary Figure 4.** *Net benefit analysis of the best performing models per modelling approach compared to a treat all and treat none strategy.*


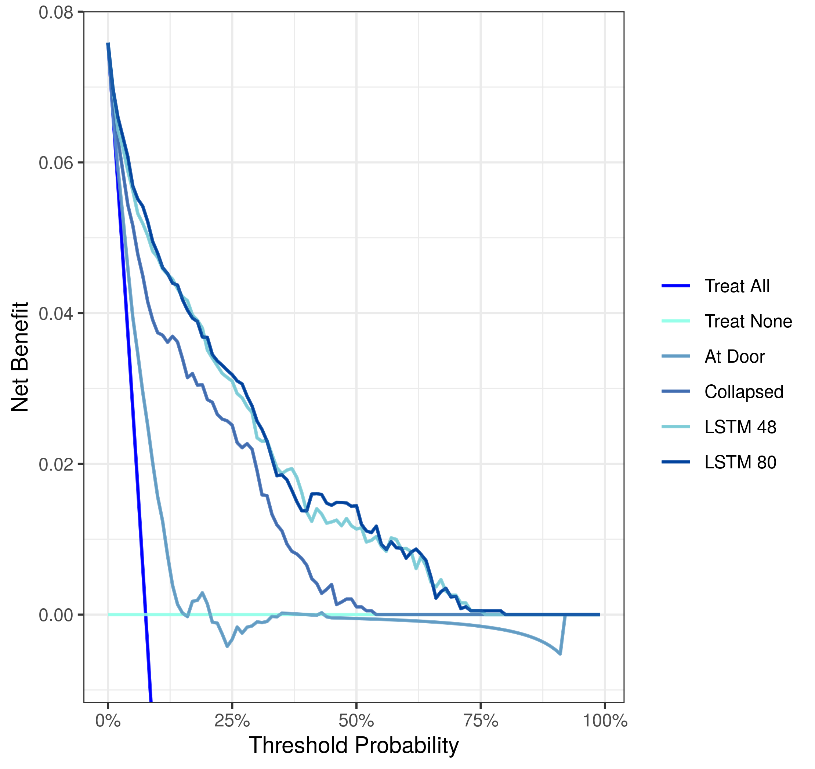


**Supplementary Figure 5.** *Feature importance for best performing models per modelling approach.*


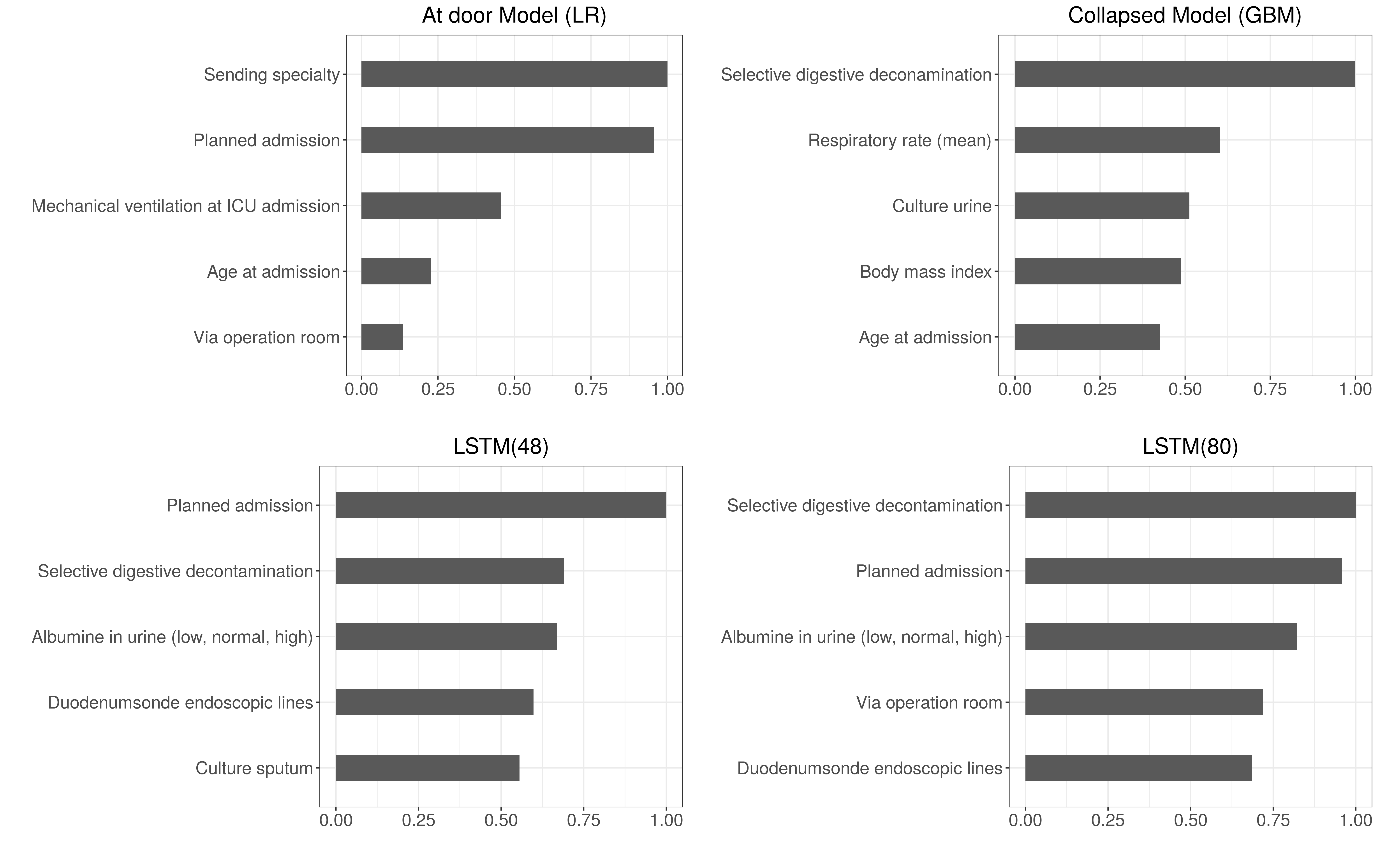

Supplement: Supplementary file 1 — Supplementary Information. [file 41598_2024_52741_MOESM1_ESM.docx]
